# Avian Influenza Ecology in North Atlantic Sea Ducks: Not All Ducks Are Created Equal

**DOI:** 10.1371/journal.pone.0144524

**Published:** 2015-12-17

**Authors:** Jeffrey S. Hall, Robin E. Russell, J. Christian Franson, Catherine Soos, Robert J. Dusek, R. Bradford Allen, Sean W. Nashold, Joshua L. TeSlaa, Jón Eínar Jónsson, Jennifer R. Ballard, Naomi Jane Harms, Justin D. Brown

**Affiliations:** 1 USGS National Wildlife Health Center, Madison, WI, United States of America, 53711; 2 Environment Canada, Science & Technology Branch, Saskatoon, Saskatchewan, Canada, S7N 5B4; 3 Maine Department of Inland Fisheries and Wildlife, Bangor, ME, United States of America, 04401; 4 University of Iceland, Snæfellsnes Research Centre, Stykkishólmur, Iceland 245; 5 Southeastern Cooperative Wildlife Disease Study, University of Georgia, Athens, GA, United States of America, 30602; 6 Department of Veterinary Pathology, Western College of Veterinary Medicine, University of Saskatchewan, Saskatoon, Saskatchewan, Canada, S7N 5B4; Linneaus University, SWEDEN

## Abstract

Wild waterfowl are primary reservoirs of avian influenza viruses (AIV). However the role of sea ducks in the ecology of avian influenza, and how that role differs from freshwater ducks, has not been examined. We obtained and analyzed sera from North Atlantic sea ducks and determined the seroprevalence in those populations. We also tested swab samples from North Atlantic sea ducks for the presence of AIV. We found relatively high serological prevalence (61%) in these sea duck populations but low virus prevalence (0.3%). Using these data we estimated that an antibody half-life of 141 weeks (3.2 years) would be required to attain these prevalences. These findings are much different than what is known in freshwater waterfowl and have implications for surveillance efforts, AIV in marine environments, and the roles of sea ducks and other long-lived waterfowl in avian influenza ecology.

## Introduction

Wild birds, primarily waterfowl (Anseriformes), as well as gulls and shorebirds (Charadriiformes) are considered the primary natural reservoirs of avian influenza virus (AIV) [1]. Therefore the majority of AIV surveillance efforts have focused on these taxa, especially freshwater dabbling ducks at migratory staging areas in autumn, and shorebirds during spring migration at Delaware Bay, USA [2, 3]. These are locations and times where large numbers of birds are readily accessible and also when high levels of AIV activity have historically been found. For example, large numbers of immunologically naïve, juvenile ducks congregate on freshwater lakes and ponds in preparation for southward migratory movements and AIV infection rates in these birds can be as high as 70% [4].

In contrast, there is little data available to investigate the ecology of influenza in marine environments. For example, the dynamics of AIV in sea ducks, including eiders (genera *Somateria* and *Polysticta*), long-tailed ducks (*Clangula hyemalis*), and scoters (genus *Melanitta*), has been studied much less frequently compared to freshwater waterfowl [2, 5–9]. Sea ducks are relatively long-lived birds that spend the majority of their lives in marine and estuarine environments and are typically more difficult to capture and sample than freshwater waterfowl. In general, sufficient numbers of sea ducks are only available for surveillance sampling at specific times of the year such as during nesting or their annual molt when access to birds is logistically easier. Wilson et al. [9] examined Alaskan wild waterfowl, including sea ducks, and found high prevalence of antibodies to AIV nucleoprotein in several eider species, with concomitant low prevalence of active viral infection. Ramey et al. [10] also found little evidence of virus infection in wintering Alaskan sea ducks. These findings suggest that AIV ecology is different in these waterfowl populations than in freshwater waterfowl. The lack of data makes it difficult to compare avian influenza ecology in sea ducks and freshwater ducks, their different environments and life histories, and geographical regions.

In addition, the overwhelming amount of available data regarding AIV in sea ducks comes from the North Pacific, specifically from Alaska, as a result of studies involved in defining the risks of virus movement into North America from East Asia [11, 12]. Little attention has been given to sea duck populations in the North Atlantic that could also be a route of intercontinental virus movement. The objectives of this study were to 1) compile and examine any existing, unpublished sea duck AIV surveillance data from the North Atlantic region, 2) test archived serum samples from North Atlantic sea ducks for historical exposure to AIV, 3) collect and analyze additional serologic and virologic samples from North Atlantic sea ducks at times of the year that have not been adequately studied, and 4) compare these data with existing surveillance data from other wild bird populations and geographic regions, as well as with data from experimental challenge studies in a model sea duck species. These results will help to determine whether influenza biology in this group of birds is fundamentally different than in other types of waterfowl, to better characterize AIV activity in marine environments, and to better tailor future surveillance efforts for this understudied group of waterfowl.

## Materials and Methods

### Sample acquisition

**Fig 1:**
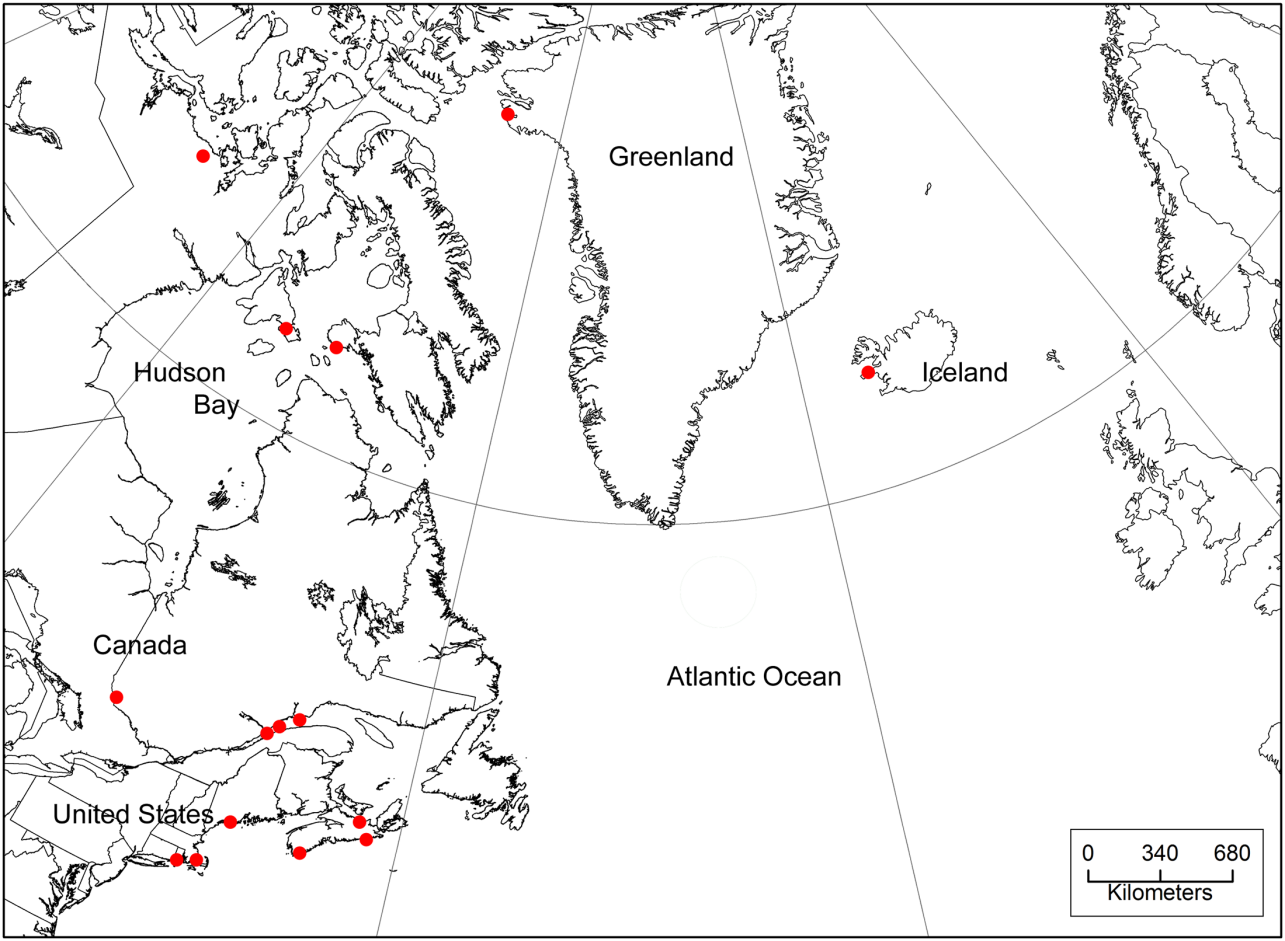
Sampling locations of sea ducks in the North Atlantic region.

This research was conducted under approval of the U.S.Geological Survey National Wildlife Health Center’s Animal Care and Use Committee, protocol number #EP090325, in strict accordance with guidelines set forth in the U.S. Governments Animal Welfare Act and the National Institutes of Health Office of Laboratory Animal Welfare. Permits for the capture and sampling of wild birds were issued by the Icelandic Institute of Natural History (Permit Numbers 368 and 403). All sampling specifically for this study occurred on public lands under the authority of the US Fish and Wildlife Service. Permits to ship collected cloacal swab and sera samples to the United States (US) were obtained from the Icelandic Institute of Natural History, the US Department of Agricultural, and the US Fish and Wildlife Service. No CITES (Convention on International Trade in Endangered Species) protected species were sampled. Acquiring archived sera and swabs, or sampling hunter harvested birds does not require Animal Care and Use Committee approval. Archived North Atlantic (Canada, Greenland, Iceland, Northeastern USA) sea duck sera were acquired from a variety of sources including the United States Geological Survey National Wildlife Health Center (NWHC), Environment Canada, and the University of Georgia. Sera were obtained from black scoter (*Melanitta nigra*), surf scoter (*Melanitta perspicillata*), white-winged scoter (*Melanitta fusca*), long-tailed duck (*Clangula hyemalis*), common eider (*Somateria mollissima*), and king eider (*Somateria spectabilis*). In addition, cloacal swab samples were collected from sea ducks on the Atlantic coast of Canada in the summers of 2007 and 2009–12 by researchers from the Canadian Wildlife Health Cooperative and Environment Canada, from Greenland (2009) by the High Arctic Institute, and from Iceland (2010–2013) by the NWHC and the University of Iceland, Snæfellsnes Research Centre (Fig 1).

In Maine, during November and December of 2011–2013, combined oral and cloacal swabs and sera were obtained from hunter harvested sea ducks. In May 2012, combined oral and cloacal swabs and sera were collected from nesting female common eiders in Maine by researchers from the NWHC and the US Fish and Wildlife Service. All swabs were obtained using Dacron tipped applicators that were placed in cryovials containing viral transport media, and stored in liquid nitrogen vapor shippers (-150°C) until transport to the NWHC where they were stored at -80°C until testing. When possible, whole blood was obtained from hunter harvested birds by cardiac puncture or from the body cavity. Blood was collected from live birds by jugular or brachial venipuncture. The cellular components were separated from serum by centrifugation in serum separator vials (Sarstedt, Newton, NC), and sera were transported and stored as described above. All swab and sera samples were obtained from adult birds.

### Serological analysis

Sea duck sera were analyzed on a Biotek EL808 ELISA reader using the IDEXX FlockChek* MultiS-Screen blocking ELISA (IDEXX Laboratories, Westbrook, ME) according to the manufacturer’s instructions.

### Real time-reverse transcription polymerase chain reaction (RT-PCR) and virus isolation

Viral RNA was extracted from cloacal and/or oral swabs taken from sea ducks using the MagMAX^™^-96 AI/ND Viral RNA Isolation Kit (Ambion, Austin, TX) following the manufacturer’s procedures. Real time RT-PCR was performed using the published procedures, primers, and probe of Spackman et al. [12] designed to detect the influenza matrix gene. RT-PCR assays were performed on a Strategene Mx3005P thermocycler using reagents provided in the Qiagen OneStep^®^ RT-PCR kit. Virus isolation was attempted on all swab samples exhibiting RT-PCR Ct values. Virus isolation was performed in embryonating chicken egg culture according to the methods described by Woolcock [13] and influenza subtypes determined by genomic sequencing. For surveillance data, samples with RT-PCR Ct values of ≤38 were considered positive for the presence of influenza virus genetic material. A less stringent Ct threshold was used to err on the side of more virus detection as opposed to the typical Ct value threshold of 35 used in typical surveillance efforts [14].

### Acquisition, care, and housing of common eiders

Common eider eggs were collected on islands off the coast of Maine, and transported to the National Wildlife Health Center (NWHC) where they were incubated (37°C, 50% humidity) until hatching. Hatchlings were housed in Biosafety level 3 isolation rooms and provided food and water ad libitum and plastic tubs of fresh water to swim in. The bird’s diet consisted of commercial waterfowl starter for 2 weeks post hatching at which time they were transitioned onto sea duck maintenance diet (Mazuri Feeds, Shoreview, MN).

### Experimental infection of common eiders with avian influenza viruses

Eider ducklings were divided into cohorts and inoculated intranasally (0.1 mL) and intrachoannally (0.9 mL) with 10^5.6^ EID_50_/mL of low pathogenic avian influenza isolate A/Mallard/Alberta/274/1979 (H3N8), or 10^6.6^ EID_50_/mL of A/long-tailed duck/Maine/295/2011 (H3N8), that were diluted in brain heart infusion broth. The inocula titers were calculated in embryonating chicken eggs using the method of Reed and Muench [15]. Two birds were mock inoculated and housed separately as controls. After inoculation, birds were weighed daily and monitored as needed to ascertain health status.

### Sampling

Daily oral and cloacal swabs were collected from experimentally challenged common eiders using Dacron tipped applicators, placed in viral transport media, and stored at -80°C. Blood samples were collected by jugular venipuncture on 0, 7 and 14 days post-inoculation (DPI) and the cellular components separated from serum by centrifugation in serum separator tubes and stored at -30°C. Water samples (2mL) were collected daily from the plastic ponds prior to replacing the water with fresh, clean tap water, and stored at -80°C until analysis. Shedding rates were measured for 14 days post infection. We conducted a linear regression with shedding rate as the response variable [16], and compared 6 models using Akaike Information Criteria (AIC) model selection to select the best model of eider shedding rates [17]. Variables included swab type (oral vs. cloacal), virus isolate (mallard vs. long-tailed duck), and days post infection as both a linear and quadratic term to capture the peak in shedding rates at the mid-point of the time series. Analyses were conducted in R using the “glm” package [18]

### Evaluating factors associated with seropositivity

We evaluated geographical and temporal factors associated with seropositivity in eiders, using logistic regression where a seropositive duck = 1 and seronegative duck = 0 [16]. All analyses were again conducted in R [17]. We evaluated three models, a model with location as a four level factor variable (Nunavut, Maine +Nova Scotia, Iceland, Rhode Island + Massachusetts), a model with season as a four-level factor variable, Spring (March 22-June 21; Summer (June 22-Sept 21) and Fall (Sept 22- Dec 21) and Winter (Dec 22-March. 21), and a model including both location and season. We used AIC to distinguish between models and select the best fit model [17].

### Estimation of antibody half-life

To estimate AIV antibody life span, we assumed a stable population at equilibrium for susceptible (S), infectious (I), and seropositive (R) individuals (i.e. *dS/dt*, *dI/dt*, and *dR/dt* = 0). We used the proportion of seropositive and infectious individuals in eider populations from our study to solve the following series of equations [19]:

dS/dt=m(1−S)−bSI+rR

dI/dt=bSI−(m+g)I

dR/dt=gI−(m+r)R

Where; m = mortality rate as well as recruitment rate (assumes stable population)

b = infection rate

g = seroconversion rate; all infected birds seroconvert thus b = g

r = seroreversion rate (antibody lifespan)

Low pathogenic avian influenza virus infection in eiders lasts for approximately one week (see experimental infection results) at which time they develop antibodies to the internal nucleoprotein (NP) as measured by the bELISA described above (seroconvert). Thus, the rate of infection (b) equals the seroconversion rate (g) for a population at equilibrium. We then obtain the equation: r = (I_e_/R_e_)g-m: where I_e_ and R_e_ are the proportions of the population that are infectious and seropositive at equilibrium. In our situation g = 1 (i.e. the infectious period lasts 1 week) and no mortality results from infection, therefore r = (I_e_/R_e_)-m.

We assessed the sensitivity of our antibody life estimates to variance in estimated seropositive and infection rates by estimating mean profile likelihood confidence intervals from our observed data. In addition, we estimated the minimum and maximum seropositive and infection rates by season and location and used those values to determine minimum and maximum possible values for antibody persistence.

## Results

### Detection of avian influenza antibodies in North Atlantic sea ducks

Because the majority of the data was derived from common eiders, we compared the seasonal and geographical differences in those populations separately from other sea duck species. Serological analysis of North Atlantic sea duck sera is summarized in Table 1 for common eiders and Table 2 for all other sea duck species. Overall, the mean seropositive rate across all populations and all seasons was 61% (95% C.I. 58–63%). Common eiders (n = 1550) had a seroprevalence of 61.5% (95% C.I. 59–64%) while all other species combined (scoter sp., long-tailed duck; n = 37) had 32% (95% C.I. 19–48%) seroprevalence.

**Table 1:** Detection of avian influenza antibodies in North Atlantic common eiders.

Location	Year	Season	No. Sampled	No. Seropositive (%)
Maine	2004	S	77	44 (57.1, 95%C.I. 46.0–67.6)
Nunavut	2007	Sp	90	47 (52.2, 95%C.I. 42.0–62.4)
Nunavut	2007	S	96	48 (50.0, 95%C.I. 40.1–60.0)
Nunavut	2008	Sp	73	41 (56.2, 95%C.I. 44.7–67.2)
Nunavut	2008	S	46	19 (41.3, 95%C.I. 27.8–55.7)
Nunavut	2009	Sp	23	9 (39.1, 95%C.I. 21.2–59.4)
Nunavut	2009	S	74	32 (43.2, 95% C.I. 32.3–54.6)
Nunavut	2010	Sp	49	26 (53.1, 95% C.I. 39.2–66.6)
Nunavut	2011	S	101	82 (81.2, 95% C.I. 72.8–88.0)
Maine	2011	Sp	225	161 (71.6, 95% C.I. 65.4–77.2)
Maine	2011	F	27	21 (77.8, 95% C.I. 60.0–90.5)
RI, MA	2011	F	87	43 (49.4, 95% C.I. 39.0–59.8)
Maine	2012	Sp	198	140 (70.7, 95% C.I. 64.1–76.8)
Maine	2012	F	69	40 (58.0, 95% C.I. 46.2–69.2)
Maine	2012	W	22	18 (81.8, 95% C.I. 62.8–94.0)
RI, MA	2012	W	71	40 (56.3, 95% C.I. 44.7–67.5)
MA	2012	Sp	22	14 (63.6, 95% C.I. 42.8–81.4)
Iceland	2012	S	38	31 (81.6, 95% C.I. 67.4–91.6)
Nova Scotia	2012	Sp	87	47 (54, 95% C.I. 43.5–64.2)
Iceland	2013	Sp	21	13 (61.9, 95% C.I. 40.7–80.4)
Maine	2013	F	40	27 (67.5, 95% C.I. 52.2–80.6)
Maine	2013	W	14	10 (71.4, 95% C.I. 45.5–90.1)
Totals			1550	953 (61.5, 95% C.I. 59.0–63.9)

Sampling season: Sp = Spring (Mar. 22-June 21); S = Summer (June 22-Sept. 21); F = Fall (Sept 22.-Dec. 21); W = Winter (Dec. 22-March 21) Rhode Island (RI), Massachusetts (MA)

**Table 2:** Detection of avian influenza antibodies in North Atlantic sea ducks other than common eiders.

Species	Location	Year	Season	No. Sampled	No. Seropositive (%)
Black Scoter	Rhode Island	2010	F	14	3 (21.4, 95% C.I 5.8–46.7)
White-winged Scoter	Rhode Island	2010	F	2	1 (50.0, 95% C.I. 3.8–96.2)
Surf Scoter	Rhode Island	2010	F	1	1 (100.0, 95% C.I. 2.5–1.0)
White-winged Scoter	Maine	2011	F	1	0 (0.0, 95% C.I. 0.0–86.2)
Surf Scoter	Maine	2011	F	2	1 (50.0, 95% C.I. 3.8–96.2)
Long-tailed Duck	Maine	2012	F	1	1 (100.0, 95% C.I. 2.5–1.0)
Black Scoter	Maine	2012	F	3	0 (0.0, 95% C.I. 0.0–59.6)
White-winged Scoter	Maine	2012	F	4	1 (25.0, 95% C.I. 2.0–72.3)
White-winged Scoter	Maine	2012	W	2	1 (50.0, 95% C.I. 3.8–96.2)
Surf Scoter	Maine	2012	W	1	0 (0.0, 95% C.I. 0.0–86.2)
Surf Scoter	Maine	2013	F	1	1(100.0, 95% C.I. 2.5–1.0)
White-winged Scoter	Maine	2013	F	2	2 (100.0, 95% C.I. 2.9–1.0)
Black Scoter	Maine	2013	F	3	0 (0.0, 95% C.I. 0.0–59.6)
Totals				37	12 (32.4, 95% C.I.18.9–48.3)

Sampling season: Sp = Spring (Mar. 22-June 21); S = Summer (June 22-Sept. 21); F = Fall (Sept 22.-Dec. 21); W = Winter (Dec. 22-March 21)

Our best model of eider seroprevalence according to AIC model selection criteria was a model with geographic location only indicating that geographical differences existed in the prevalence of AIV antibodies (Table 3). Neither of the other two models was within 2 AIC points of this model. Parameter estimates indicated that eiders sampled in Nunavut (z = -2.81, p = 0.005), Rhode Island and Massachusetts (z = -2.75, p = 0.006) were less likely to test positive for influenza antibodies than eiders sampled in Iceland. There was no statistical difference between the overall seroprevalence of eiders captured in Nova Scotia and Maine versus those sampled in Iceland (z = -1.21, p = 0.227, Table 2). The highest seroprevalence we observed in eiders occurred in summer 2011 at Nunavut, (81%, 95% C.I. 73–88%), summer 2012 in Iceland, (81%, 95% C.I. 67–92%), and Maine in the winter of 2012, (82%, 95% C.I.). The lowest seroprevalences, occurred in Nunavut in the spring of 2009 (39%, 95% C.I. 21.2–59.4), and the summer of 2008 (41.3%, 95% C.I. 27.8–55.7). Although Maine was the only location where sampling took place during all seasons of the year, sampling in Maine was not consistent over all of the years.

**Table 3:** Parameter estimates from the best model of common eider seroprevalence as selected by Akaike Information Criteria (n = 1550).

Parameter Estimate	Estimate	Std. Error	z value	Pr(>|z|)
Intercept	1.08	0.30	3.60	<0.001
Maine + Nova Scotia vs. Iceland	-0.37	0.31	-1.21	0.227
Nunavut vs. Iceland	-0.87	0.31	-2.81	0.005
RI+ Massachusetts vs. Iceland	-0.92	0.33	-2.75	0.006

### Detection of avian influenza virus infection in North Atlantic sea ducks

In contrast to the prevalence of AIV antibodies, detection of AIV infection in North Atlantic sea ducks was low. In general, relatively few virus infections were detected with a total of 12 RT-PCR positive swab samples detected from common eiders (Table 4) and another 3 positive samples from other sea duck species (Table 5). The overall sea duck virus prevalence was 0.4% (95% C.I. 0.2–0.6%), 0.3% in common eiders (95% C.I. 0.2–0.5%), and 0.6% (95% C.I. 0.1%-1.6%) in the other species. Interestingly, the majority of the AIV positive samples came from just two sampling seasons, 6 positive common eider samples from summer 2010 on their breeding grounds in Nunavut, Canada (1.1% prevalence, 95% C.I. 0.4–2%), and from 2 common eiders, a black scoter and two long-tailed ducks in Maine during the fall/winter of 2011 (1.3% prevalence, 95% C.I. 0.3–3.3%). At all other locations and/or times combined, the overall virus prevalence was less than 0.1% and other than the sea duck sampling in Maine 2011, no AIV infection was found in any species other than in common eiders.

**Table 4:** RT-PCR detection of avian influenza virus RNA in North Atlantic common eiders.

Year	Location	Season	No. Sampled	No. Positive (%)
2006–10	Maine	Sp	177	0 (0.0, 95% C.I. 0.0–1.8)
2006–10	Maine	S	272	0 (0.0, 95% C.I. 0.0–1.2)
2006–10	Maine	F	10	0 (0.0, 95% C.I. 0.0–27.0)
2006–10	Maine	W	25	0 (0.0, 95% C.I. 0.0–12.3)
2006–10	Massachusetts	Sp	4	0 (0.0, 95% C.I. 0.0–51.0)
2006–10	Massachusetts	W	22	0 (0.0, 95% C.I. 0.0–13.8)
2006–10	Rhode Island	Sp	1	0 (0.0, 95% C.I. 0.0–86.2)
2006–10	Rhode Island	F	13	0 (0.0, 95% C.I. 0.–21.8)
2006–10	Rhode Island	W	13	0 (0.0, 95% C.I. 0.0–21.8)
2007	Nunavut	S	456	2 (0.4, 95% C.I. 0.0–1.3)
2009	Greenland	S	14	0 (0.0, 95% C.I. 0.0–20.5)
2010	Iceland	S	35	0 (0.0, 95% C.I. 0.0–9.0)
2010	Greenland	S	17	0 (0.0, 95% C.I. 0.0–17.3)
2009	New Brunswick	S	68	0 (0.0, 95% C.I. 0.0–4.8)
2009	Nova Scotia	S	81	0 (0.0, 95% C.I. 0.0–4.0)
2009	Nunavut	Sp	45	0 (0.0, 95% C.I. 0.0–7.1)
2009	Nunavut	S	390	0 (0.0, 95% C.I. 0.0–0.8)
2010	Nunavut	Sp	167	6 (3.6, 95% C.I. 1.4–7.1)
2010	Nunavut	S	304	0 (0.0, 95% C.I. 0.0–1.1)
2010	Nova Scotia	S	68	0 (0.0, 95% C.I. 0.0–4.8)
2011	Nunavut	Sp	188	0 (0.0, 95% C.I. 0.0–1.7)
2011	Nunavut	S	113	0 (0.0, 95% C.I. 0.0–2.9)
2011	Nova Scotia	S	57	0 (0.0, 95% C.I. 0.0–5.7)
2011	Maine	F	238	2 (0.8, 95% C.I. 0.0–2.6)
2012	Maine	Sp	99	0 (0.0, 95% C.I. 0.0–3.3)
2012	Maine	F	214	1 (0.5, 95% C.I. 0.0–0.2)
2012	Maine	W	42	0 (0.5, 95% C.I. 0.0–7.5)
2012	Iceland	Sp	58	0 (0.5, 95% C.I. 0.0–5.6)
2013	Iceland	Sp	1	1 (100.0, 95% C.I. 2.5–1.0)
2013	Maine	F	94	0 (0.0, 95% C.I. 0.0–3.5)
2013	Maine	W	39	0 (0.0, 95% C.I. 0.0–8.1)
Totals			3212	12 (0.4, 95% C.I. 0.2–0.6)

Sampling season: Sp = Spring (Mar. 22-June 21); S = Summer (June 22-Sept. 21); F = Fall (Sept 22.-Dec. 21); W = Winter (Dec. 22-March 21)

**Table 5:** RT-PCR detection of avian influenza virus RNA in North Atlantic sea ducks, other than common eiders.

Species	Year	Location	Season	No. Sampled	No. Positive (%)
King Eider	2009–11	Nunavut	S	89	0 (0.0, 95% C.I. 3.5–1.0)
Long-tailed Duck	2009	Greenland	S	3	0 (0.0, 95% C.I. 0.0–59.5)
Black Scoter	2010	Quebec	Sp	55	0 (0.0, 95% C.I. 0.0–5.9)
White-winged Scoter	2010	Quebec	S	58	0 (0.0, 95% C.I. 0.0–5.6)
Surf Scoter	2010	Quebec	S	83	0 (0.0, 95% C.I. 0.0–4.0)
Long-tailed Duck	2011	Maine	F	113	2 (1.8, 95% C.I. 0.0–5.4)
White-winged Scoter	2011	Maine	F	17	0 (0.0, 95% C.I. 0.0–17.3)
Black Scoter	2011	Maine	F	5	1 (20, 95% C.I. 0.0–62.8)
Surf Scoter	2011	Maine	F	10	0 (0.0, 95% C.I. 0.0–27.0)
Long-tailed Duck	2012	Maine	F	55	0 (0.0, 95% C.I. 0.0–5.9)
Long-tailed Duck	2012	Maine	W	12	0 (0.0, 95% C.I. 0.0–23.3)
White-winged Scoter	2012	Maine	F	42	0 (0.0, 95% C.I. 0.0–7.6)
White-winged Scoter	2012	Maine	W	5	0 (0.0, 95% C.I. 0.0–44.6)
Black Scoter	2012	Maine	F	24	0 (0.0, 95% C.I. 0.0–12.8)
Black Scoter	2012	Maine	W	3	0 (0.0, 95% C.I. 0.0–59.5)
Surf Scoter	2012	Maine	F	6	0 (0.0, 95% C.I. 0.0–39.4)
Surf Scoter	2012	Maine	W	1	0 (0.0, 95% C.I. 0.0–86.2)
Black Scoter	2013	Maine	F	6	0 (0.0, 95% C.I. 0.0–39.4)
Black Scoter	2013	Maine	W	1	0 (0.0, 95% C.I. 0.0–86.2)
Surf Scoter	2013	Maine	F	12	0 (0.0, 95% C.I. 0.0–23.3)
Surf Scoter	2013	Maine	W	1	0 (0.0, 95% C.I. 0.0–86.2)
White-winged Scoter	2013	Maine	F	21	0 (0.0, 95% C.I. 0.0–14.4)
Long-tailed Duck	2013	Maine	F	59	0 (0.0, 95% C.I. 0.0–5.6)
Long-tailed Duck	2013	Maine	W	2	0 (0.0, 95% C.I. 0.0–71.0)
Totals				683	3 (0.4, 95% C.I. 0.0–1.1)

Sampling season: Sp = Spring (Mar. 22-June 21); S = Summer (June 22-Sept. 21); F = Fall (Sept 22.-Dec. 21); W = Winter (Dec. 22-March 21)

A total of four AIV were isolated from North Atlantic sea duck swab samples, all collected in Maine during the Fall/Winter of 2011. All were different subtypes indicating that multiple AIV were regionally circulating in sea duck populations at that season: A/common eider/Maine/505/2011(H12N5), Genbank Accession numbers CY149612-19; A/black scoter/Maine/276/2011(H10N7), CY149572-79; A/common eider/Maine/270/2011(H1N1), CY149564-71; A/long-tailed duck/Maine/295/2011(H3N8), CY149588-95. BLAST analysis of all 8 gene segments of each AIV isolate showed that the H1N1, H10N7 and H3N8 subtype viruses were most similar to North American waterfowl lineage viruses, including isolates from freshwater ducks sampled in 2011 elsewhere within the same region (New Brunswick, Nova Scotia, Maine). Interestingly, genes from both the H1N1 (PA gene) and the H10N7 (HA) isolates had RNA segments highly similar (99% and 98% respectively) to North American waterfowl viruses isolated from gulls in Iceland [20]. Additionally, 3 gene segments (PB1, NP, NS) from the H12N7 isolate were similar to AIV obtained from a Steller’s eider (*Polysticta stelleri*) and a king eider in Alaska as well as other Pacific Coast North American waterfowl. The PB2 gene of this virus was most (97%) similar to Eurasian lineage AIV. These data indicate that AIV populations frequently reassort and have interconnections that span local, regional, hemispheric and transcontinental distances, and also may reflect the circumpolar distribution of sea duck populations.

### Experimental challenge of common eiders with A/Mallard/Alberta/274/1979 (H3N8)

Following challenge with a H3N8 virus isolate from a freshwater duck, all five eiders in this cohort became infected and excreted moderate amounts of virus, both orally and cloacally (Table 6). Discounting the DPI 1 oral excretion data which likely represented residual inocula, the amount of virus was higher in cloacal swabs than oropharyngeal (OP) swabs, and peaked on DPI 3–4 (range 10^2.36^–10^4.09^ EID_50_/mL). The duration of virus shedding was 5–7 days after inoculation and no bird excreted virus longer than 7 DPI (not shown). None of the challenged or control birds exhibited any weight loss, behavioral abnormalities, or any other overt clinical signs of infection.

**Table 6:** Excretion of virus from common eiders experimentally inoculated with 10^5.6^ EID_50_ of Influenza A/Mallard/Alberta/274/1979 (H3N8).

Oral excretion.								
Bird ID	DPI 0	1	2	3	4	5	6	7
53	No Ct	No Ct	-0.26*	-0.24	0.23	2.31	No Ct	0.14
56	No Ct	2.45	1.80	0.90	1.59	1.42	0.41	0.00
59	No Ct	4.16	1.01	2.70	1.16	0.76	No Ct	No Ct
63	No Ct	2.00	2.68	1.67	1.75	2.03	0.53	-0.77
67	No Ct	5.05	3.47	1.90	1.89	1.41	No Ct	0.94
Cloacal excretion.								
53	No Ct	2.85	1.90	3.66	3.21	3.12	0.45	3.66
56	No Ct	No Ct	2.43	2.63	3.29	0.74	0.55	-0.09
59	No Ct	-0.25	0.67	0.36	2.36	1.52	-0.54	0.00
63	No Ct	0.53	1.65	3.34	2.19	2.01	0.96	0.13
67	No Ct	0.85	0.56	1.37	4.09	1.44	0.88	No Ct

*Values are based on quantitative RT-PCR analysis and expressed as Log_10_ EID_50_/ml.

### Experimental challenge of common eiders with A/long-tailed duck/Maine/295/2011(H3N8)

Experimental challenge with the long-tailed duck influenza H3N8 virus isolate resulted in all birds becoming infected and excreting virus (Table 7). Cloacal excretion was higher than oral and lasted from 5–7 days post inoculation, however, the levels of virus shedding were generally lower in this cohort than those infected with the mallard isolate. No viral RNA was detected in any swab sample after 7 DPI and none of the challenged birds exhibited clinical signs of disease or infection.

**Table 7:** Excretion of virus from common eiders experimentally inoculated with 10^6.6^ EID_50_ of Influenza A/long-tailed duck/Maine/295/2011 (H3N8).

Oral excretion.								
Bird ID	DPI 0	1	2	3	4	5	6	7
51	No Ct	0.92*	0	1.06	0.17	0.32	-1.35	-0.8
54	No Ct	1.47	-0.05	3	1.89	-0.22	No Ct	No Ct
57	No Ct	2.9	1.15	0.4	0.37	0.23	-0.61	-1.3
61	No Ct	-0.11	0.42	1.32	0.59	0.03	0.02	-1.11
64	No Ct	2.73	0.65	1.69	1.58	0.21	-0.11	-0.15
69	No Ct	1.38	0.06	1.53	0.21	0.35	-1.73	-0.61
Cloacal excretion.								
51	No Ct	0.42	0.66	1.14	1.43	-0.69	No Ct	No Ct
54	No Ct	-0.99	1.23	2.64	1.98	1.07	2.11	-0.58
57	No Ct	1.59	0.68	1.34	0.9	1.72	0.64	2.24
61	No Ct	2.5	0.34	2.03	1.22	1.22	No Ct	No Ct
64	No Ct	2.27	1.8	1.93	2.69	3.11	-0.6	-0.3
69	No Ct	3.6	2.6	1.69	2.85	3.15	2.62	2.6

*Values are based on quantitative RT-PCR analysis and expressed as Log_10_ EID_50_/ml.

Parameter estimates from regression models of the two virus challenge cohorts indicated that shedding rates increased and then decreased as a function of time (Table 8), with no statistically significant differences between swab types, or species from which the inocula were isolated.

**Table 8:** Parameter estimates of the best model as selected by AIC for virus shedding in experimentally challenged common eiders.

Parameter Estimate	Mean	SE	T-value	P-value
Intercept	-3.83	0.5	-7.7	<0.0001
DPI	3.29	0.33	9.93	<0.0001
DPI*DPI	-0.43	0.05	-9.43	<0.0001
Swab type (oral vs. cloacal)	0.66	0.7.	0.94	0.347
DPI*Swab	-0.63	0.47	-1.35	0.18
DPI*DPI*Swab	0.06	0.06	0.94	0.346

### Detection of influenza viruses in environmental samples from experimentally challenged common eiders

The tubs of water that the birds were allowed to swim and bath in, were sampled daily prior to having the water replaced with fresh, clean water. Analysis of these samples by RT-PCR revealed that moderate quantities of both the mallard and the long-tailed duck AI H3N8 viruses were detected in the environmental samples based on Ct values of ~32 (Table 9).

**Table 9:** RT-PCR analysis of environmental samples* from common eiders experimentally inoculated with Influenza A H3N8 viruses.

Virus Isolate		
Day Post-inoculation	Mallard	Long-tailed duck
DPI 1	29.66^1^	33.15
DPI 2	32.25	32.26
DPI 3	31.92	31.42
DPI 4	32.3	31.91
DPI 5	35.97	NS
DPI 6	NS	35.98

*Water samples taken from plastic pools in which birds were allowed to swim/bathe ad libitim.

^1^Values expressed are Ct values of RT-PCR assays.

NS- no sample collected

### Serological analysis of experimentally challenged common eiders\

The serological response of common eiders to experimental AIV challenge is summarized in Table 10. All virus challenged common eiders seroconverted by DPI 7 based on bELISA results, however they had relatively weak DPI 14 HI titers to the mallard and long-tailed duck viruses (1:10–1:20). Thus we can assume that sea ducks serologically respond to influenza virus infection in a manner consistent with our data from field studies, and that the bELISA performs well with sea duck sera.

**Table 10:** Serological response of common eiders experimentally challenged with Influenza A H3N8 viruses.

		DPI 0		DPI 7		DPI 14		
Virus Inoculum	ID	S/N Ratio*	Result	S/N Ratio	Result	S/N Ratio	Result	HI Titer
Control	60	0.925	N	0.852	N	0.777	N	<1:10^1^
	68	0.898	N	0.843	N	0.770	N	<1:10
Mallard/Alberta/274/1979	53	0.915	N	0.247	P	0.275	P	<1:10
	56	0.868	N	0.282	P	0.310	P	1:10
	59	0.865	N	0.241	P	0.300	P	1:10
	63	0.813	N	0.251	P	0.344	P	<1:10
	67	0.889	N	0.211	P	0.364	P	1:10
Long-tailed Duck/ME/295/2011	51	0.906	N	0.316	P	0.488	P	1:10
	54	0.876	N	0.141	P	0.111	P	1:10
	57	0.899	N	0.169	P	0.138	P	1:20
	61	0.891	N	0.315	P	0.340	P	1:10
	64	0.815	N	0.212	P	0.094	P	1:20
	69	0.824	N	0.208	P	0.152	P	1:10

*Determined using IDEXX Multi S bELISA; S/N ratios <0.50 considered positive (P) for AI antibodies; N—negative.

^1^Hemagglutination inhibition (HI) assays conducted using same virus as inocula according to methods of Beard (1989). Negative control sera were <1:10 to both viruses.

### Avian influenza antibody persistence estimation in common eiders

Estimated mean seroprevalence across eiders populations was 62% (95% C.I. 59–64%) (Table 1). Our estimated infectious proportion of the eider population was 0.4% (95% C.I. 0.2–0.6%). Using band recovery data from adult female common eiders in eastern North America, Kremetz et al. [21] calculated an estimated annual survival rate of 0.873 and average life span of 7.36 years, respectively. Thus, an average annual survival rate of 0.873 indicates a weekly mortality rate of 0.0024. Using our estimates of Ie, Re, and m, we conclude that the seroreversion rate (r), or antibody lifespan, is = 0.0025, leading to an estimated half-life of 171 weeks (3.2 years). Using the confidence intervals of 59–64% for mean seropositive eiders and 0.2%-0.6% mean infection rates, we get a range of values for the antibody life from a minimum of 89 weeks (1.7 years; for 59% seropositive, 0.6% infected population) to a maximum of 956 weeks (18.4 years; for a 64% seropositive, 0.2% infected population).

## Discussion

Based on our experimental challenge data, common eiders are efficiently infected (100% infected) by both freshwater duck and sea duck influenza virus isolates, excrete moderate amounts of virus, with infection lasting approximately one week at which time all birds seroconverted. The wild sea duck populations that we sampled exhibited high seroprevalence rates but low virus prevalence, regardless of location or time of year. In contrast, dabbling duck populations have high cyclical virus infection rates that are driven by seasonal concentrations of immunologically naïve, recently hatched young [1].

There are basic differences in these host taxa natural histories that may help explain differences in AIV biology. Sea ducks have longer lifespans, breed at older ages, reproduce at lower rates than freshwater counterparts, and have different migration and seasonal congregation patterns than freshwater waterfowl. The fact that sea ducks reside in marine and estuarine environments raises questions regarding whether the fecal/oral paradigm of AIV transmission observed in dabbling ducks and shorebirds, manifests similarly in sea ducks. Water depth, tidal action, dilution, currents, waves, temperature, and salinity can all potentially affect AIV stability and accessibility and hence the likelihood of virus transmission and infection of sea ducks [22–24].

We did, however, uncover evidence of episodic waves of AIV infection in sea duck populations. Of the 15 RT-PCR positive swab samples out of over 4000 tested, 6 came from Nunavut, Canada eiders in the summer of 2010 and 5 came from various sea duck species sampled in Maine in the fall and winter of 2011. We were unable to isolate viruses from the Canadian samples but, based on sequence data, multiple AIV subtypes were circulating and infecting sea ducks in coastal Maine in 2011 including H3N8, H10N1, H10N7, and H12N5 viruses that had genetic relationships with viruses isolated in 2011 from Iceland, Canada, and Alaska. There was also a mortality event in New England harbor seals that fall and winter from a mammalian adapted avian influenza H3N8 virus [25, 26]. In all other sampling periods, very few AIV infections were uncovered in sea duck populations in Maine, Nunavut, or elsewhere. Why these years resulted in more AIV activity in sea ducks than other years is unknown. Interestingly, the highest seroprevalences we observed were in these same locations the year following the virus outbreaks (81.2% seropositive in Summer 2011 Nunavut; 81.8% in Winter 2012 Maine). This is consistent with our estimates of AIV antibodies lasting more than one year after exposure. Future studies should investigate whether climactic factors, such as changes in ocean temperatures, are contributing to these sporadic, epizootic events.

The relatively high AIV seroprevalence observed in sea duck populations may be a function of low virus exposure rates but long-lived circulating antibodies. Sea ducks have relatively long lifespans, therefore, even though virus exposure rates are low, serological evidence of exposure can accumulate over time to the observed levels if antibodies remain detectable for relatively long periods. It is generally accepted that longer-lived species invest more biological resources in acquired immune responses than short-lived species [27]. This may be the case with sea ducks compared to freshwater dabbling ducks that have much shorter lifespans. In an experimental trial conducted in mallard ducks (*Anas platyrhynchos*), researchers found that all inoculated birds became ELISA seropositive within 1 week, but that after 7 weeks post inoculation, half of the birds (6/12) had reverted to seronegative status [28]. This is similar to the findings of Tolf et al [29] in naturally infected mallards. In contrast, Hoye et al. [30] estimated that AIV antibodies in pink-footed geese (*Anser brachyrhynchus*), a relatively long-lived species, are detectable for up to one year, which is more similar to our calculated antibody lifespan in eiders of 3.4 years.

Unstructured sampling regimes make it difficult to make large scale inferences from our data. To acquire adequate sample numbers, most surveillance studies have relied on accessing hunter harvested birds, breeding females on nests, and/or molting flightless birds. Therefore, the timeframe at which samples can potentially be collected is limited. In addition we were unable to obtain samples from juvenile birds; data from which would provide a direct comparison to juvenile freshwater ducks. We also divided our data based on calendar season instead of biological season. Obtaining archived samples from a vast region such as the North Atlantic, with asynchronous populations, we were unable to categorize the data temporally based on biological markers. Therefore, although our data summaries show no seasonal effects on influenza virus infection in sea ducks, we cannot dismiss the possibility that sea duck populations sampled at different times of the year or at different life stages may reveal different patterns in viral infections.

Though several aspects of the avian influenza transmission cycle, such as annual recruitment (hatch) pulse, serial AIV infections, and cross subtype protection, were not factors included in the calculations, our estimates of antibody lifespan strongly suggest that detectable antibodies in eiders last longer than has been previously described in other avian species. Clearly, immune responses of reservoir hosts are important to understand, and the length of these responses is a key component of any transmission model. Experimental infection studies that measure antibody levels over extended time frames will be needed to adequately answer this question.

Much attention has been focused on AIV transmission, movement, and genetic reassortment in avian populations in Alaska and Eastern Russia as this would be a region where highly pathogenic avian influenza H5N1, or other viruses, would likely be introduced into North America by migratory wild birds [11, 31–33]. More recently, the North Atlantic region has been shown to also be important in AIV ecology and intercontinental virus movement [20, 34–37]. The role of sea ducks in the ecology of avian influenza is largely unknown. We provide virological and serological data showing that these birds have low virus prevalence and high seroprevalence in all populations examined, similar to the findings of Wilson et al. [9]. Based on these data and known life histories of common eiders we calculated that AIV antibody lifespan in common eiders must be longer than one year to reach the levels found in these wild populations. Sea ducks in particular, and North Atlantic bird populations in general, have been drastically under-represented in influenza surveillance efforts. Although our data did not permit detailed comparison of marine versus freshwater species, differences in AIV dynamics in these habitats, including subtype constellations, transmission mechanisms, and potential movement of viruses between the marine and terrestrial ecosystems warrant additional study
